# Antibiotic Resistance Can Be Enhanced in Gram-Positive Species by Some Biocidal Agents Used for Disinfection

**DOI:** 10.3390/antibiotics8010013

**Published:** 2019-02-01

**Authors:** Günter Kampf

**Affiliations:** Institute for Hygiene and Environmental Medicine, University Medicine Greifswald, 17475 Greifswald, Germany; guenter.kampf@uni-greifswald.de

**Keywords:** biocide, cross-resistance, cross-tolerance, antibiotics, antiseptic stewardship

## Abstract

Some biocidal agents used for disinfection have been described to enhance antibiotic resistance in Gram-negative species. The aim of this review was therefore to evaluate the effect of 13 biocidal agents at sublethal concentrations on antibiotic resistance in Gram-positive species. A MEDLINE search was performed for each biocidal agent on antibiotic tolerance, antibiotic resistance, horizontal gene transfer, and efflux pump. Most data were reported with food-associated bacterial species. In cells adapted to benzalkonium chloride, a new resistance was most frequently found to ampicillin (seven species), cefotaxime and sulfamethoxazole (six species each), and ceftazidime (five species), some of them with relevance for healthcare-associated infections such as *Enterococcus faecium* and *Enterococcus faecalis*. With chlorhexidine, a new resistance was often found to imipenem (ten species) as well as cefotaxime, ceftazidime, and tetracycline (seven species each). Cross-resistance was also found with triclosan and ceftazidime (eight species), whereas it was very uncommon for didecyldimethylammonium chloride or hydrogen peroxide. No cross-resistance to antibiotics has been described after low level exposure to glutaraldehyde, ethanol, propanol, peracetic acid, octenidine, povidone iodine, sodium hypochlorite, and polyhexanide. Preference should be given to disinfectant formulations based on biocidal agents with a low or no selection pressure potential.

## 1. Introduction

In some parts of the world, the incidence of nosocomial infections caused by vancomycin-resistant enterococci (VRE) is increasing [1,2,3]. The cost of nosocomial VRE infections is substantial [4]. Prior antibiotic treatment has been described to be a relevant risk factor among patients in hematology and oncology [5]. However, it is currently unknown if other antimicrobial compounds such as specific biocidal agents used in the immediate patient environment for disinfection may have a similar effect on the microbial flora on inanimate surfaces or the skin of patients or healthcare workers.

Most users of disinfectants do not expect that some of the biocidal agents in the disinfectants can cause antibiotic resistance themselves, although it has been shown recently in various Gram-negative species, especially during low level exposure [6]. There is still a lack of awareness on this topic in the infection control community [7]. The aim of the review is therefore to summarize data on the development of antibiotic resistance and tolerance, induction of antibiotic resistance genes, changes of horizontal gene transfer, and the effect on common efflux pump genes in Gram-positive species caused by low level exposure to some commonly used biocidal agents. The method is described in Appendix A.

## 2. Benzalkonium Chloride

### 2.1. Antibiotic Tolerance or Resistance after Low Level Biocide Exposure

One study described an increase of tolerance of *Lactobacillus pentosus* to some antibiotics such as chloramphenicol (up to 500-fold), teicoplanin (up to 340-fold), ampicillin (up to 100-fold), or tetracycline (up to 80-fold) after exposure to a sublethal concentration of benzalkonium chloride (1 mg/L). The adaptive potential of *Leuconostoc pseudomesenteroides* was much lower (Table 1).

Three studies were found describing a new resistance to various antibiotics after exposure to variable but increasing sublethal concentrations of benzalkonium chloride. In seven species it was a resistance to ampicillin, in six species a resistance to sulfamethoxazole or cefotaxime, in five species a resistance to ceftazidime, in three species a resistance to tetracycline, and in one species a resistance to ciprofloxacin (Table 2). Among the species, some have a major relevance for healthcare-associated infections, such as *Enterococcus faecalis* or *Enterococcus faecium*.

### 2.2. Induction of Common Efflux Pumps

Low level exposure of four *Listeria monocytogenes* isolates susceptible to benzalkonium chloride was able to increase efflux pump activity in three of them [12]. In a *Listeria monocytogenes* wild type outbreak strain, the emrE efflux function was upregulated 49.6-fold [13].

Another interesting observation was made in the Netherlands. The epidemiology of *Listeria monocytogenes* meningitis has changed over 25 years. Peak incidence rates were observed in neonates (0.61 per 100,000 live births) and older adults (peak at 87 years old; 0.53 cases per 100,000 population of the same age). Whereas most clonal complexes decreased over time, one of them increased significantly increased (clonal complex 6; from 2% to 26%). The emrC efflux transporter has been shown to be associated with the emergence of this clonal complex 6 in the Netherlands. The emrC gene encodes an efflux protein that pumps quaternary ammonium compounds out of the cell. In addition, it increases the capacity to form a biofilm, both resulting in benzalkonium chloride tolerance. Isolates harboring emrC were less susceptible to amoxicillin and gentamicin compared with isolates without emrC. Benzalkonium chloride is extensively used in the foodprocessing industry as a disinfectant agent. Reduced susceptibility to benzalkonium chloride may be an explanation for the increasing incidence of clonal complex 6 isolates in the Netherlands between 1985 and 2014 [14].

### 2.3. Additional Findings

A general adaptation to benzalkonium chloride by Gram-positive bacteria cannot be expected. When *Enterococcus faecalis* and *Staphylococcus aureus* were exposed on agar over 14 passages of 4 days each to increasing concentrations of benzalkonium chloride, both increases and decreases in antibiotic susceptibility were found, but its effect was typically small relative to the differences observed among microbicides. Susceptibility changes resulting in resistance were not observed in this study [15]. In 1632 clinical *Staphylococcus aureus* isolates, a correlation of susceptibility profiles of at least 0.4 was found to benzalkonium chloride and the quinolones, beta-lactams, and macrolides [16]. Additionally, a MIC value > 2 mg/L for benzalkonium chloride was associated with multidrug antibiotic resistance in *Staphylococcus aureus*, as demonstrated in 1632 human clinical *Staphylococcus aureus* isolates from different geographical regions [17]. Another study with 200 *Listeria monocytogenes* isolates, however, showed no association between resistance to benzalkonium chloride and antibiotics [18]. And in five lactic acid bacteria strains with a resistance to benzalkonium chloride, no cross-resistance to other antimicrobials was found except for gentamicin [19].

## 3. Chlorhexidine Digluconate

### 3.1. Antibiotic Tolerance or Resistance after Low Level Biocide Exposure

One study described that in several *Staphylococcus aureus* isolates the MIC values for antibiotics such as tetracycline, amikacin, or gentamicin can increase up to 512-fold after 14 days (Table 3).

Two other studies showed that exposure to variable but increasing sublethal concentrations of chlorhexidine digluconate can enhance a new resistance to various antibiotics, most frequently to imipenem (10 species), ceftazidime, tetracycline, or cefotaxime (seven species each), sulfamethoxazole (five species), and ampicillin (three species). Among the species, some have a major relevance for healthcare-associated infections, such as *Enterococcus faecalis* or *Enterococcus faecium* (Table 4).

### 3.2. Effect on Antibiotic Resistance Genes

VanA-type vancomycin resistance gene expression was increased in a vanA *Enterococcus faecium* (≥10-fold increase of vanHAX encoding) [23].

### 3.3. Increase of Horizontal Gene Transfer

In two strains and three derivates of *Bacillus subtilis*, no increase of transfer of the mobile genetic element Tn916, a conjugative transposon, was observed [24]. No significant reduction of plasmid transfer frequency was detected in *Staphylococcus aureus* [25].

### 3.4. Additional Findings

When healthcare workers used a soap based on 2% chlorhexidine digluconate, they had a relative risk of 1.92 and 1.50 of having their hand colonized with a *Staphylococcus epidermidis* resistant to oxacillin or resistant to gentamicin, respectively [26]. In *Staphylococcus warneri*, the relative risk for rifampicin resistance was even 7.22 [26]. An analysis of 301 *Staphylococcus aureus* isolates from three African countries showed a significant association between specific resistance genes for biocidal agents (sepA, mepA, norA, lmrS, qacAB, smr) and resistance to antibiotics [27]. Recent data show that exposure of vancomycin-resistant *Enterococcus faecium* to chlorhexidine digluconate for only 15 min upregulates the vanA-type vancomycin resistance gene (vanHAX) and genes associated with reduced daptomycin susceptibility (liaXYZ) [28]. In another VRE strain, a subpopulation with reduced daptomycin susceptibility including significant alterations in membrane phospholipids was detected after 21 days of chlorhexidine digluconate exposure at various concentrations [22]. In a study, 120 clinical MRSA isolates were exposed to various concentrations of chlorhexidine digluconate (range: 2.5–40 mg/mL), which were allowed to dry in a glass bottle. Possible changes in the susceptibility to eight antibiotics (ampicillin, tetracycline, vancomycin, gentamicin, oxacillin, cefotaxime, cefuroxime, ciprofloxacin) were determined. The MICs of cefotaxime, vancomycin, gentamicin, cefuroxime, and oxacillin increased in the epidemic MRSA strain 16 following 48 h of residue drying. There were also increases in the MICs of all tested antibiotics for NCTC 6571, a *Staphylococcus aureus* susceptible strain, following exposure to chlorhexidine residues that had been drying for 48 h (compared with the MICs for the strain before exposure). The increases in the MICs of all tested antibiotics for the susceptible control *Staphylococcus aureus* strain following exposure to surface dried chlorhexidine residues is of interest, as it suggests that the use of chlorhexidine in the hospital environment may be linked to increased resistance to antibiotics in previously susceptible strains [29]. An analysis of 247 nosocomial *Staphylococcus aureus* isolates revealed that smr-positive *Staphylococcus aureus* isolates (44.0%) were more often resistant to methicillin, ciprofloxacin, and/or clindamycin [30]. The isolates positive for qacA/B (33.6%) had more often a vancomycin MIC of ≥2 mg/L [30]. An analysis of multiresistance plasmids found in 280 staphylococcal isolates from diverse geographical regions from the 1940s to the 2000s suggested that enormous selective pressure has optimized the content of certain plasmids despite their large size and complex organization [31]. In addition, plasmid pSAJ1 from a methicillin- and gentamicin-resistant strain of *Staphylococcus aureus* conferred resistance to chlorhexidine and in addition to kanamycin, gentamicin, tobramycin, amikacin, benzalkonium chloride, acriflavine, and ethidium bromide [32]. In 1632 clinical *Staphylococcus aureus* isolates, a correlation of susceptibility profiles of at least 0.4 was found to chlorhexidine digluconate and ciprofloxacin [16]. An analysis of 1632 human clinical *Staphylococcus aureus* isolates from different geographical regions showed that a MIC value > 2 for chlorhexidine digluconate is associated with multidrug antibiotic resistance in *Staphylococcus aureus* [17].

## 4. Triclosan

### 4.1. Antibiotic Tolerance or Resistance after Low Level Biocide Exposure

One study described that in quite a few *Lactobacillus pentosus* isolates the MIC values for antibiotics such as teicoplanin, chloramphenicol, or ampicillin can increase up to 340-fold after exposure to 1 mg/L triclosan over 48 h. Adaptive changes of antibiotic susceptibility was substantially less pronounced in *Leuconostoc pseudomesenteroides* (Table 5).

Three other studies showed that exposure to variable but increasing sublethal concentrations of triclosan can enhance increased tolerance or a new resistance to various antibiotics, most frequently to ceftazidime (eight species), sulfamethoxazole and cefotaxime (six species each), and ampicillin (two species). Among the species, at least *Enterococcus faecium* has relevance for healthcare-associated infections (Table 6).

In dust, a significant positive association between the ubiquitous antimicrobial triclosan and the relative abundance of the antibiotic resistance gene erm(X) was observed; a 23S rRNA methyltransferase implicated resistance to several antibiotics [36].

### 4.2. Increase of Horizontal Gene Transfer

In *Staphylococcus aureus*, an additional sh-fabI allele derived from *Staphylococcus haemolyticus* was detected. Detection of sh-fabI as a novel resistance mechanism with high potential for horizontal gene transfer demonstrates for the first time that a biocide could exert a selective pressure able to drive the spread of a resistance determinant in a human pathogen [37]. In addition, both the introduction of a plasmid expressing the saFabI gene or a missense mutation in the chromosomal saFabI gene led to triclosan resistance in *Staphylococcus aureus* [38].

### 4.3. Additional Findings

The triclosan resistance mechanisms are the same types of mechanisms involved in antibiotic resistance, and some of them account for the observed cross-tolerance with antibiotics in laboratory isolates. Therefore, there is a link between triclosan and antibiotics, and the widespread use of triclosan-containing antiseptics and disinfectants may indeed aid in the development of microbial resistance, in particular cross-resistance to antibiotics [39]. A triclosan-tolerant *Staphylococcus aureus* strain did not reveal a cross-resistance to any of the 33 tested antibiotics [40]. In addition, in 1632 clinical *Staphylococcus aureus* isolates no cross-resistance was detected to any clinically relevant antibiotic [16]. Finally, an investigation of indoor dust revealed that dust with high triclosan content contained increased Gram-positive species with diverse drug resistance capabilities, whose pangenomes were enriched for genes encoding osmotic stress responses, efflux pump regulation, lipid metabolism, and material transport across cell membranes [41].

## 5. Didecyldimethylammonium Chloride

One study showed that didecyldimethylammonium chloride (DDAC)-MICs were positively correlated with several other antibiotic MICs (e.g., chloramphenicol in *Enterococcus faecalis*), and increased DDAC-MICs were statistically linked to high-level resistance to streptomycin in enterococci [42]. In *Listeria monocytogenes*, resistance to tetracycline and streptomycin was described in one of 31 strains [43]. Overall, however, exposure of two species (*Enterococcus faecalis*, *Staphylococcus aureus*) over 14 passages of 4 days each to increasing DDAC concentrations on agar was associated with both increases and decreases in antibiotic susceptibility, but its effect was typically small relative to the differences observed among microbicides. Susceptibility changes resulting in resistance were not observed [15].

## 6. Hydrogen Peroxide

In *Staphylococcus aureus* NCIMB 9518, an unstable resistance to ciprofloxacin was detected after exposure to sublethal concentrations of hydrogen peroxide [44]. Another finding was described in *Bacillus subtilis* cells. The transfer of the mobile genetic element Tn916, a conjugative transposon and the prototype of a large family of related elements, was not increased by exposure to 0.002% hydrogen peroxide for up to 2 h [24].

## 7. Polyhexanide

Exposure of *Enterococcus faecalis* and *Staphylococcus aureus* over 14 passages of 4 days each to increasing polyhexanide concentrations on agar was associated with both increases and decreases in antibiotic susceptibility, but its effect was typically small relative to the differences observed among microbiocides. Susceptibility changes resulting in resistance were not observed [15]. No other data were found.

## 8. Sodium Hypochlorite

In 1632 clinical *Staphylococcus aureus* isolates, no correlation of susceptibility profiles was found to sodium hypochlorite and any clinically relevant antibiotic [16].

## 9. Other Biocidal Agents

No cross-tolerance or cross-resistance to antibiotics has so far been described in Gram-positive bacterial species for glutaraldehyde, ethanol, propanol, peracetic acid, octenidine, and povidone iodine.

## 10. Discussion

Biocidal agents used for chemical disinfection in healthcare or food processing are one of many elements to limit the spread of antibiotic resistant bacteria. Most users of chemical disinfectants do not expect that these biocidal agents may cause antibiotic resistance themselves. Triclosan is a prominent example. It was used for decades in antimicrobial soaps in the domestic setting in the US and considered to be safe and effective [45]. In 2016, however, 19 active ingredients including triclosan were banned by the US Food and Drug Administration for antimicrobial soaps used at home by the general population [46]. The decision was justified by associated risks including antibiotic resistance and a lack of a health benefit (page 61107): “A risk must be balanced by a demonstration—through studies that demonstrate a direct clinical benefit (i.e., a reduction of infection)—that the product is superior to washing with non-antibacterial soap and water in reducing infection.” One of the associated risks is obviously the possibility of bacterial species developing antibiotic resistance.

Some Gram-positive species are able to exhibit a tolerance or even resistance to various antibiotics after exposure to sublethal concentrations of a few biocidal agents used for disinfection, especially benzalkonium chloride, chlorhexidine digluconate, and triclosan. Other biocidal agents, however, did not enhance antibiotic resistance. Based on these finding, it seems only logical to review the use of biocidal agents for disinfection and to prefer those agents with lower or no potential to cause antimicrobial tolerance or resistance, as proposed before for Gram-negative species [6]. It is also advisable to review disinfectant product groups (e.g., alcohol-based hand rubs). They are typically the first choice for healthcare workers to decontaminate their hands with the aim of reducing healthcare-associated infections [47]. Additional biocidal agents in alcohol-based hand rubs, such as chlorhexidine digluconate or octenidine, for example, have no evidence for any health benefit [48]. As a consequence, their use should be reviewed. Preference should be given to products without additional biocidal agents as long as they have equivalent user acceptability and efficacy for hand disinfection (“antiseptic stewardship”) [49]. The Commission for Hospital Hygiene and Infection Control (KRINKO) at the Robert Koch-Institute, Berlin, Germany, has therefore published in its guideline that alcohol-based hand rubs with persistent biocidal agents cannot be recommended [50].

Antibiotic resistance is a major concern, with an estimated 671,689 infections caused by antibiotic-resistant bacteria in Europe in 2015, of which 63.5% were associated with healthcare [51]. A major limitation of this review is that most data demonstrating an adaptive change of Gram-positive species were reported with food-associated species, although the typical nosocomial pathogens *Enterococcus faecium* and *Enterococcus faecalis* were also found. It is therefore difficult to say if these findings can also be expected with typical healthcare-associated Gram-positive bacterial species.

## 11. Conclusions

Antibiotic resistance may occur after exposure of various Gram-positive species, mainly *Enterococcus* spp., to sublethal concentrations of some biocidal agents such as benzalkonium chloride, chlorhexidine, or triclosan. With an increase of nosocomial VRE infections, these antiseptic agents should be used only for applications with a proven health benefit. General preference should be given to biocidal agents without or with a low selection pressure, assuming that their antimicrobial activity, material compatibility, and user safety is at least as good for the intended use.

## Figures and Tables

**Table 1:** Gram-positive species with increased antibiotic tolerance after various types of low level exposure (<MIC value) to benzalkonium chloride (BAC).

Species	Strain(s)	Type of Exposure	MIC Increase (BAC)	Antibiotic(s)	MIC Increase (Antibiotic)	Reference
*Lactobacillus pentosus*	Seven strains from naturally fermented Aloreña green table olives	48 h at 1 mg/L	-	Ampicillin Chloramphenicol Ciprofloxacin Teicoplanin Tetracycline Trimethoprim Clindamycin Erythromycin Streptomycin	1-fold–100-fold ^1^ 2-fold–500-fold ^1^ 2-fold–14-fold ^1^ 1-fold–340-fold ^1^ 2-fold–80-fold ^1^ 1-fold–15-fold ^1^ None ^1^ None ^1^ None ^1^	[8]
*Leuconostoc pseudomesenteroides*	Strain from naturally fermented Aloreña green table olives	48 h at 1 mg/L	-	Ciprofloxacin Chloramphenicol Tetracycline Ampicillin Clindamycin Erythromycin Streptomycin Teicoplanin Trimethoprim	3-fold ^1^ 2-fold ^1^ 2-fold ^1^ None ^1^ None ^1^ None ^1^ None ^1^ None ^1^ None ^1^	[8]

^1^ microdilution method (mg/L); MIC = minimum inhibitory concentration.

**Table 2:** Gram-positive species with a new phenotypic antibiotic resistance after various types of low level exposure (<MIC value) to benzalkonium chloride (BAC).

Species	Strain(s)	Type of Exposure	MIC Increase (BAC)	Antibiotic(s)	Pre-Value	Post-Value	Category	Reference
*Bacillus cereus*	Five biocide-sensitive strains from organic foods	Several passages with gradually higher concentrations	10-fold– 200-fold	Ampicillin Sulfamethoxazole Cefotaxime	- - -	64 (2) ^1^ 1024 (2) ^1^ 128 (1) ^1^	R R R	[9]
*Bacillus licheniformis*	Two biocide-sensitive strains from organic foods	Several passages with gradually higher concentrations	25-fold– 50-fold	Ceftazidime Cefotaxime	- -	64 (1) ^1^ 128 (1) ^1^	R R	[9]
*Bacillus* spp.	Four biocide-sensitive strains from organic foods	Several passages with gradually higher concentrations	4-fold– 25-fold	Sulfamethoxazole Ampicillin Cefotaxime	- - -	1024 (2) ^1^ 64 (1) ^1^ 128 (1) ^1^	R R R	[9]
*Enterococcus casseliflavus*	Two biocide-sensitive strains from organic foods	Several passages with gradually higher concentrations	10-fold– 20-fold	Ampicillin	-	32 (1) ^1^	R	[9]
*Enterococcus durans*	Biocide-sensitive strain from organic foods	Several passages with gradually higher concentrations	4-fold	Ampicillin	-	32 ^1^	R	[9]
*Enterococcus faecalis*	Two biocide-sensitive strains from organic foods	Several passages with gradually higher concentrations	5-fold– 50-fold	Ceftazidime Cefotaxime Sulfamethoxazole	- - -	64 (2) ^1^ 128 (1) ^1^ 1024 (1) ^1^	R R R	[9]
*Enterococcus faecium*	Thirteen biocide-sensitive strains from organic foods	Several passages with gradually higher concentrations	4-fold– 50-fold	Ampicillin, Cefotaxime Ciprofloxacin Tetracycline	- - - -	16 or 32 (7) ^1^ 64 (3) ^1^ 8 (2) ^1^ 32 (1) ^1^	R R R R	[9]
*Enterococcus* spp.	Six biocide-sensitive strains from organic foods	Several passages with gradually higher concentrations	4-fold– 35-fold	Ampicillin Cefotaxime Ceftazidime Sulfamethoxazole	- - - -	16 or 32 (3) ^1^ 64 or 128 (2) ^1^ 64 (2) ^1^ 1024 (1) ^1^	R R R R	[9]
*Listeria monocytogenes*	25 strains from food or food production	Several passages with gradually higher concentrations	2-fold– 5-fold	Ampicillin Cefotaxime Cephalotin Chloramphenicol Ciprofloxacin Erythromycin Kanamycin Tetracycline	0.25–2 ^1^ 2–16 ^1^ 2–32 ^1^ 1–8 ^1^ 0.5–4 ^1^ 0.125–0.5 ^1^ 1–4 ^1^ 0.5–1 ^1^	0.25–2 ^1^ 8–64 ^1 *^ 16–64 ^1 *^ 1–8 ^1^ 1–8 ^1^ 0.125–0.5 ^1^ 1–4 ^1^ 0.5–1 ^1^	- - - - - - - -	[10]
*Listeria monocytogenes*	Four isolates sensitive to BAC	2–3 w at variable concentrations	4-fold– 6-fold	Gentamicin Kanamycin	2.25–4.5 ^1^ 6.25–12.5 ^1^	1.4–5.5 ^1^ 6.25–25 ^1^	- -	[11]
*Staphylococcus saprophyticus*	Five biocide-sensitive strains from organic foods	Several passages with gradually higher concentrations	10-fold– 200-fold	Sulfamethoxazole Ceftazidime Ampicillin Tetracycline	- - - -	1024 (3) ^1^ 64 (3) ^1^ 64 (2) ^1^ 16 (1) ^1^	R R R R	[9]
*Staphylococcus* spp.	Four biocide-sensitive strains from organic foods	Several passages with gradually higher concentrations	2-fold– 150-fold	Sulfamethoxazole Ampicillin Ceftazidime Tetracycline	- - - -	1024 (3) ^1^ 32 or 64 (3) ^1^ 64 (1) ^1^ 32 (1) ^1^	R R R R	[9]

^1^ microdilution method (mg/L); “-” = no information; R = resistant; () = number of strains or isolates; * increase in all strains.

**Table 3:** Gram-positive species with increased antibiotic tolerance after various types of low level exposure (<MIC value) to chlorhexidine digluconate (CHG).

Species	Strain(s)	Type of Exposure	MIC Increase (CHG)	Antibiotic(s)	MIC Increase (Antibiotic)	Reference
*Staphylococcus aureus*	ATCC 25923 and 14 clinical isolates	14 d at various sublethal concentrations	4-fold–6-fold (6 isolates)	Ciprofloxacin Tetracycline Gentamicin Amikacin Cefepime Meropenem	4-fold–64-fold (6) ^1^ 4-fold–512-fold (15) ^1^ 4-fold–512-fold (8) ^1^ 16-fold–512-fold (11) ^1^ 8-fold–64-fold (11) ^1^ 8-fold–64-fold (9) ^1^	[20]

^1^ microdilution method (mg/L); () = number of strains or isolates.

**Table 4:** Gram-positive species with a new phenotypic antibiotic resistance after various types of low level exposure (<MIC value) to chlorhexidine digluconate (CHG).

Species	Strain(s)	Type of Exposure	MIC Increase (CHG)	Antibiotic(s)	Pre-Value	Post-Value	Category	Reference
*Bacillus cereus*	Four biocide-sensitive strains from organic foods	Several passages with gradually higher concentrations	6-fold– 16-fold	Imipenem Sulfamethoxazole Ampicillin Tetracycline	- - - -	16 (4) ^1^ 1024 (2) ^1^ 64 (1) ^1^ 32 (1) ^1^	R R R R	[21]
*Bacillus licheniformis*	Two biocide-sensitive strains from organic foods	Several passages with gradually higher concentrations	4-fold– 10-fold	Imipenem Cefotaxime Tetracycline	- - -	16 (2) ^1^ 64 (1) ^1^ 32 (1) ^1^	R R R	[21]
*Bacillus* spp.	Four biocide-sensitive strains from organic foods	Several passages with gradually higher concentrations	4-fold– 8-fold	Imipenem Sulfamethoxazole Cefotaxime Ceftazidime	- - - -	16 (4) ^1^ 1024 (4) ^1^ 64 (1) ^1^ 64 (1) ^1^	R R R R	[21]
*Enterococcus casseliflavus*	Three biocide-sensitive strains from organic foods	Several passages with gradually higher concentrations	8-fold– 20-fold	Imipenem Cefotaxime Tetracycline	- - -	16 (3) ^1^ 64 (1) ^1^ 32 (1) ^1^	R R R	[21]
*Enterococcus durans*	Biocide-sensitive strain from organic foods	Several passages with gradually higher concentrations	10-fold	Imipenem Ampicillin	- -	16 ^1^ 64 ^1^	R R	[21]
*Enterococcus faecalis*	Biocide-sensitive strain from organic foods	Several passages with gradually higher concentrations	10-fold	Imipenem Ceftazidime	- -	16 ^1^ 64 ^1^	R R	[21]
*Enterococcus faecium*	Nine biocide-sensitive strains from organic foods	Several passages with gradually higher concentrations	2-fold– 16-fold	Imipenem Tetracycline Ampicillin Cefotaxime Ceftazidime	- - - - -	16 (9) ^1^ 16 or 32 (4) ^1^ 32 or 64 (2) ^1^ 128 (1) ^1^ 64 (1) ^1^	R R R R R	[21]
*Enterococcus faecium*	Clinical VRE strain	21 d at various concentrations	4-fold	Daptomycin	2	3–6 ^1, 2^	-	[22]
*Enterococcus* spp.	Six biocide-sensitive strains from organic foods	Several passages with gradually higher concentrations	2-fold– 10-fold	Imipenem Ceftazidime Sulfamethoxazole Cefotaxime Tetracycline	- - - - - -	16 (6) ^1^ 64 (5) ^1^ 1024 (5) ^1^ 64–128 (4) ^1^ 16 (3) ^1^	R R R R R	[21]
*Staphylococcus saprophyticus*	Four biocide-sensitive strains from organic foods	Several passages with gradually higher concentrations	2-fold– 10-fold	Ceftazidime Imipenem Sulfamethoxazole Cefotaxime Tetracycline	- - - - -	64 (4) ^1^ 16 (2) ^1^ 1024 (2) ^1^ 128 (2) ^1^ 16 (1) ^1^	R R R R R	[21]
*Staphylococcus xylosus*	Biocide-sensitive strain from organic foods	Several passages with gradually higher concentrations	4-fold	Ceftazidime Imipenem Sulfamethoxazole Cefotaxime Tetracycline	- - - - -	64 ^1^ 16 ^1^ 1024 ^1^ 128 ^1^ 16 ^1^	R R R R R	[21]
*Staphylococcus* spp.	Three biocide-sensitive strains from organic foods	Several passages with gradually higher concentrations	4-fold– 10-fold	Ceftazidime	-	64 (1) ^1^	R	[21]

^1^ microdilution method (mg/L); ^2^ subpopulation; “-” = no information; R = resistant; () = number ofstrains or isolates. VRE: vancomycin-resistant enterococci.

**Table 5:** Gram-positive species with increased antibiotic tolerance after various types of low level exposure (<MIC value) to triclosan (TRI).

Species	Strain(s)	Type of Exposure	MIC Increase(TRI)	Antibiotic(s)	MIC Increase (Antibiotic)	Reference
*Lactobacillus pentosus*	Seven strains from naturally fermented Aloreña green table olives	48 h at 1 mg/L	-	Ampicillin Chloramphenicol Ciprofloxacin Teicoplanin Tetracycline Trimethoprim Clindamycin Erythromycin Streptomycin	5-fold–100-fold ^1^ Up to 200-fold (6) ^1^ Up to 7-fold (6) ^1^ Up to 340-fold (5) ^1^ 2-fold–80-fold (6) ^1^ 15-fold (1) ^1^ None None None	[8]
*Leuconostoc pseudomesenteroides*	Strain from naturally fermented Aloreña green table olives	48 h at 1 mg/L	-	Ciprofloxacin Chloramphenicol Tetracycline Ampicillin Clindamycin Erythromycin Streptomycin Teicoplanin Trimethoprim	7-fold ^1^ 2-fold ^1^ None ^1^ None ^1^ None ^1^ None ^1^ None ^1^ None ^1^ None ^1^	[8]

^1^ microdilution method (mg/L); () = number of strains or isolates.

**Table 6:** Gram-positive species with a new phenotypic antibiotic resistance after various types of low level exposure (<MIC value) to triclosan (TRI).

Species	Strain(s)	Type of Exposure	MIC Increase (TRI)	Antibiotic(s)	Pre-Value	Post-Value	Category	Reference
*Bacillus cereus*	Five biocide-sensitive strains from organic foods	Several passages with gradually higher concentrations	3-fold– 4-fold	Sulfamethoxazole Ampicillin Cefotaxime Ceftazidime	- - - -	1024 (3) ^1^ 64 (1) ^1^ 128 (1) ^1^ 64 (1) ^1^	R R R R	[33]
*Bacillus licheniformis*	Biocide-sensitive strain from organic foods	Several passages with gradually higher concentrations	3-fold	Sulfamethoxazole Ceftazidime	- -	1024 ^1^ 64 ^1^	R R	[33]
*Bacillus* spp.	Four biocide-sensitive strains from organic foods	Several passages with gradually higher concentrations	2-fold	Sulfamethoxazole Cefotaxime Ceftazidime	- - -	1024 (2) ^1^ 128 (1) ^1^ 64 (1) ^1^	R R R	[33]
*Enterococcus casseliflavus*	Biocide-sensitive strain from organic foods	Several passages with gradually higher concentrations	2-fold	Cefotaxime	-	128 ^1^	R	[33]
*Enterococcus faecium*	Five biocide-sensitive strains from organic foods	Several passages with gradually higher concentrations	2-fold– 4-fold	Cefotaxime Ceftazidime	- -	64 (1) ^1^ 64 (1) ^1^	R R	[33]
*Enterococcus* spp.	Two biocide-sensitive strains from organic foods	Several passages with gradually higher concentrations	2-fold	Ceftazidime	-	64 (1) ^1^	R	[33]
*Lactobacillus rhamnosus*	Strain AC413	10 passages of 4 h at various concentrations	None	Metronidazole Tetracycline	500 ^1^ 5.2 ^1^	500 ^1^ 3.9 ^1^	- -	[34]
*Listeria monocytogenes*	Eight strains from different sources	4 × 24 h (1 and 4 mg/L)	-	Gentamicin	5–20 ^1^	40–160 ^1^	-	[35]
*Staphylococcus saprophyticus*	Three biocide-sensitive strains from organic foods	Several passages with gradually higher concentrations	3-fold– 5-fold	Sulfamethoxazole Cefotaxime Ceftazidime	- - -	1024 (2) ^1^ 128 (1) ^1^ 64 (1) ^1^	R R R	[33]
*Staphylococcus xylosus*	Biocide-sensitive strain from organic foods	Several passages with gradually higher concentrations	5-fold	Sulfamethoxazole Ceftazidime	- -	1024 ^1^ 64 ^1^	R R	[33]
*Staphylococcus* spp.	Two biocide-sensitive strains from organic foods	Several passages with gradually higher concentrations	2-fold– 150-fold	Sulfamethoxazole Ceftazidime Cefotaxime Ampicillin	- - - -	1024 (2) ^1^ 32 or 64 (2) ^1^ 128 (1) ^1^ 64 (1) ^1^	R R R R	[33]
*Streptococcus mutans*	NCTC 10832	10 passages of 4 h at various concentrations	None	Metronidazole Tetracycline	62.5 ^1^ 1.0 ^1^	62.5 ^1^ 2.0 ^1^	- -	[35]
*Streptococcus oralis*	NCTC 11427	10 passages of 4 h at various concentrations	1.7-fold	Metronidazole Tetracycline	62.5 ^1^ 7.8 ^1^	125 ^1^ 3.9 ^1^	- -	[35]
*Streptococcus sanguis*	NCTC 7863	10 passages of 4 h at various concentrations	None	Metronidazole Tetracycline	62.5 ^1^ 7.8 ^1^	125 ^1^ 7.8 ^1^	- -	[35]

^1^ microdilution method (mg/L); “-” = no information; R = resistant; () = number of strains or isolates.

## Appendix A

The following biocidal agents were reviewed: triclosan, benzalkonium chloride, hydrogen peroxide, glutaraldehyde, ethanol, chlorhexidine digluconate, sodium hypochlorite, didecyldimethylammonium chloride (DDAC), octenidine, peracetic acid, propanol, polyhexanide, and povidone iodine. A MEDLINE search was performed on 14 January 2019. For “horizontal gene transfer”, antibiotic, and each biocidal agent, the MEDLINE search revealed five hits for hydrogen peroxide, four hits for ethanol and triclosan, two hits for benzalkonium chloride, one hit for chlorhexidine digluconate, glutaraldehyde, sodium hypochlorite, propanol, povidone iodine, peracetic acid, and octenidine, and no hits for DDAC and polyhexanide. For “cross tolerance”, antibiotic, and each biocidal agent, the MEDLINE search revealed eight hits for ethanol, seven hits for benzalkonium chloride and hydrogen peroxide, four hits for povidone iodine, three hits for sodium hypochlorite and glutaraldehyde, one hit for propanol, polyhexanide, chlorhexidine digluconate, octenidine, and peracetic acid, and no hits for DDAC. For “cross resistance”, antibiotic, and each biocidal agent, the MEDLINE search revealed 46 hits for triclosan, 41 hits for benzalkonium chloride, 37 hits for hydrogen peroxide, 28 hits for chlorhexidine digluconate, 26 hits for ethanol, 23 hits for povidone iodine and glutaraldehyde, 12 hits for sodium hypochlorite, eight hits for peracetic acid, three hits for propanol and octenidine, two hits for polyhexanide, and one hit for DDAC. For “efflux pump”, antibiotic, and each biocidal agent, the MEDLINE search revealed 41 hits for triclosan, 38 hits for benzalkonium chloride, 15 hits for hydrogen peroxide, 14 hits for ethanol, eight hits for propanol, six hits for chlorhexidine digluconate, four hits for glutaraldehyde, two hits for sodium hypochlorite, one hit for octenidine, peracetic acid, and povidone iodine, and no hits for DDAC and polyhexanide.

Publications were included and results were extracted from them when they provided original data on an adaptive response to the exposure of Gram-positive bacteria to sublethal concentrations of the biocidal agents described above resulting in a tolerance or resistance to antibiotics including antibiotic resistance gene changes, a change of efflux pump activity, or horizontal gene transfer. Articles were excluded when they described changes in Gram-negative species, fungi, or mycobacteria. Reviews were also excluded but screened for any information within the scope of the review.
